# Retention of Pollutants Elements from Mine Tailings of Lead in Geopolymers for Construction

**DOI:** 10.3390/ma14206184

**Published:** 2021-10-18

**Authors:** Juan María Terrones-Saeta, Jorge Suárez-Macías, Ana María Castañón, Fernando Gómez-Fernández, Francisco Antonio Corpas-Iglesias

**Affiliations:** 1 Research Group TEP 222 “Materials and Mining Engineering”, Higher Polytechnic School of Linares, University of Jaen, 23700 Linares, Spain; jsuarez@ujaen.es (J.S.-M.); facorpas@ujaen.es (F.A.C.-I.); 2 Department of Mining, Topography and Structures, University of León (ESTIM), Campus de Vegazana, s.n, 24071 León, Spain; amcasg@unileon.es (A.M.C.); fgomf@unileon.es (F.G.-F.)

**Keywords:** geopolymer, mining waste, retention of potentially toxic elements, potentially toxic elements, circular economy, sustainability, construction materials

## Abstract

The construction sector is one of the most demanding sectors of raw materials in existence today. As a consequence, the extraction of these materials has a significant impact on the environment. At the same time, mining activities produce a series of wastes, in some cases with polluting elements, which must be treated to avoid pollution. Therefore, the use of mining waste for the conformation of new construction materials is an important environmental advantage, even more so when such waste is prevented from producing polluting leachates. Therefore, in this research, geopolymers are developed with mine tailings from the Linares lead mines, chemically activated with potassium hydroxide. For this purpose, different percentages of the alkaline activator were tested and the physical and mechanical properties of the conformed materials were evaluated. The analysis of the different conformed geopolymers determined the optimum percentage of potassium hydroxide for conforming the geopolymer with the best mechanical and physical properties. In addition, the concentration in the leachate of potentially contaminating chemical elements in the mining waste was estimated to be lower than those regulated by the regulations. Consequently, this research shows the development of a sustainable material for construction with mining waste and reduction of the environmental impact of traditional products.

## 1. Introduction

The development of the population’s well-being entails the consumption of large amounts of resources [1]. In addition, this development leads to the generation of enormous amounts of waste. Therefore, the currently used system of a linear economy, in which raw materials are extracted and the product is manufactured, used, and finally landfilled, is not a viable option for society today [2]. Consequently, a new circular economy has emerged based on the reuse of waste as raw material for new materials, thus avoiding the landfilling of waste, reducing the extraction of raw materials, and creating a closed material flow [3].

Based on the above, this type of circular economy can be applied to different sectors and activities. However, it is interesting to apply this new methodology in activities that consume large quantities of raw materials [4] and, at the same time, produce huge amounts of waste [5], as is the case for the construction and mining sectors.

Mining activities are essential for the development of the population [6], as they obtain resources used in different sectors. However, these mining activities produce a series of highly polluting wastes that must be properly treated to avoid impact on the environment [7,8]. In most cases these wastes are deposited in landfills; however, these wastes still have an important potential to be used in other materials.

Additionally, the construction sector is one of the most demanding in terms of raw materials that currently exists, causing significant greenhouse gas emissions [9,10]. So much so that the high consumption of raw materials such as clay means that this type of resource is being depleted, making it necessary to search for more sustainable alternative solutions [11].

Based on the above, the use of mining waste as a raw material for construction materials is a sustainable solution that should be considered [12,13]. However, for the reuse of this mining waste, it must be ensured that the potentially toxic elements existing in the waste are encapsulated in the new material [14,15], thus avoiding environmental pollution. It is therefore a very suitable solution when trying to avoid contamination of mining waste, extraction of raw materials, and landfilling of waste.

There are different materials that can contain the waste with greater or lesser viability; however, new materials formulated with waste must also be produced with less environmental impact and with similar quality to traditional products. One such new material is geopolymer [16].

Geopolymers, developed in 1978 by Joseph Davidovits [17], have been developed in various research projects as substitutes for traditional cement [18] and even ceramics [19]. This material is an inorganic polymer formed by the combination and reaction of an aluminosilicate and an alkaline activator, initially forming aluminate and silicate monomers, then oligomers, and finally geopolymers [20]. This material has excellent fire resistance [21], good mechanical strength [22], good chemical stability [23], and most importantly, it is capable of retaining potential element contaminants [24].

The aluminosilicates used for the development of geopolymers in the various published studies have mainly been waste, such as: fly ash [25,26], metakaolin [27,28], slag from metallurgical industries [29,30], glass waste [31,32], bagasse [33,34], etc. In turn, as alkaline activators [35,36], sodium hydroxide or potassium hydroxide have mainly been used.

Based on the above, this research develops geopolymers for construction with tailings from the mining district of Linares (Spain) as a source of aluminosilicate, and potassium hydroxide as an alkaline activator. In this way, the contamination produced by this mining waste from old mines deposited in the outdoors is avoided; a new construction material is developed as a substitute for cement and even ceramics; the extraction of new raw materials is avoided; and potentially toxic elements that can leach from the tailings are retained in this material.

With the aim of developing the described objective of this research, initially a physical-chemical characterisation of the waste was carried out, determining its suitability as a source of aluminosilicate and the existence of potentially toxic elements to be retained in the geopolymer. Subsequently, and only with the material from the mine tailings and potassium hydroxide, different families of geopolymers were conformed with different percentages of alkaline activator. These geopolymer families were physically and mechanically evaluated to determine the correct combination of aluminosilicate and alkaline activator. Finally, in order to check that the potentially toxic elements in the mine tailings were retained in the geopolymer matrix, leachate tests were carried out and the concentrations of contaminating elements were calculated with respect to the limits set by the regulations.

## 2. Materials and Methods

This section describes the materials and methodology, consisting of different tests, used to objectively assess the feasibility of manufacturing geopolymers from mine tailings for the retention of potentially toxic elements of the tailings and with properties suitable for use as construction materials.

### 2.1. Materials

The geopolymers of the present research have been developed with the fundamental idea of obtaining a building material composed mainly of waste. For this reason, aluminosilicate material from mine tailings from the Linares district, Spain, was used as a source of aluminosilicate. This material was activated with an alkaline activator, more specifically with potassium hydroxide. Consequently, the main materials of this research are the mine tailings, called “Collado de Lobo”, and potassium hydroxide. These materials are described in more detail in the following sections in order to be able to replicate the results and define the materials used.

#### 2.1.1. “Collado del Lobo” Mine Tailing

The mining district of Linares, located in the south of Spain, developed an intense mining activity in the period between the end of the 19th century and the 20th century. This mining activity was mainly based on the extraction and concentration of lead, derived from lead sulphides, galena, or argentiferous galena. Associated with these types of sulphides were other sulphides of copper, arsenic, zinc, which at the time of exploitation were of no economic interest and were deposited on the surface as waste. It should be noted that the aforementioned sulphides were developed by hydrothermal processes in the fissures of the existing country rock in the area, which was mainly granite or slate. These rocks, of a siliceous nature, were extracted in order to obtain lead and were also deposited on the surface, which is why they are an important source of aluminosilicates.

However, as noted above, these siliceous rocks contain other elements of little interest at the time of mining, such as zinc, arsenic, barium, copper, etc., which can cause significant environmental pollution. Furthermore, the concentration methods used, which are totally obsolete today, did not make it possible to recover all the lead in the extracted material, so there are also potentially toxic concentrations of this chemical element.

More specifically, the mine tailings waste from the Linares district has all the aforementioned components and, as a major environmental disadvantage, small particle size. This mining waste was produced by crushing the material for the concentration of lead by gravimetric methods in most cases or by flotation. The waste from these mining processes was deposited directly on the surface without any waterproofing or treatment system to avoid environmental contamination of ground or surface water, flora, fauna, etc., as environmental regulations in those past decades were almost non-existent. As a result, these large deposits of mining waste are currently exposed outdoors and produce significant environmental pollution.

In this research, in particular, a mine tailing called “Collado de Lobo” was analysed and used. This mine tailing has an approximate volume of 50,000 cubic metres and does not have any type of environmental barrier to prevent contamination.

The samples of this material, for the development of this research, were taken according to the UNE-EN 932-1 standard. Subsequently, the sample was taken to the laboratory to be dried at a temperature of 105 ± 2 °C for 24 h in order to eliminate the moisture. The existence of moisture in the waste is not detrimental to the industrial process, it must simply be considered in order to add the correct percentage of water for the geopolymerisation process. In this case, the waste was dried to avoid unknown variables that could cloud the results.

Once the sample was dry, it was divided into different subsamples for characterisation or for geopolymer conformation, according to the UNE-EN 932-2 standard. The samples for chemical characterisation were ground to a particle size of less than 100 μm. This process was carried out for the best chemical analysis of the samples due to the restrictions of the analysis equipment used. For geopolymer conformation, as well as physical characterisation tests, no grinding process was carried out, and the mine tailing sample was used directly after drying.

#### 2.1.2. Potassium Hydroxide

Potassium hydroxide was used as an alkaline activator to conform the geopolymers. This material was used instead of sodium hydroxide for two fundamental reasons: its lower economic cost in large quantities and the lower environmental impact produced by this element when neutralised. The potassium hydroxide used had a purity of 85%.

### 2.2. Methodology

The methodology of this research is composed of a series of logically ordered tests to assess the feasibility of conforming geopolymers with mining tailings that retain, in turn, the potentially toxic elements of the waste.

First, a chemical and physical characterisation of the mining waste was carried out, determining its suitability for use as a raw material for construction materials, as well as the existence of toxic elements that should be monitored. Subsequently, geopolymers were conformed with the mine tailing “Collado del Lobo” and different percentages of potassium hydroxide. In this way, the physical and mechanical properties of the geopolymers conformed could be evaluated, as well as the variation of the properties produced by the increase in the alkaline activator. Finally, the TCLP method was used to determine the concentration of potentially toxic elements in the leachate of the different families of geopolymers conformed, determining that they complied with the limits set by the EPA.

The following sections describe in greater detail the tests carried out, divided into the three main blocks of this methodology. Characterisation of the mine tailings, geopolymer conformation and testing, and evaluation of the leachates produced by the geopolymers.

#### 2.2.1. Physical and Chemical Characterisation of the Mine Tailings “Collado del Lobo”

The physical-chemical characterisation of mine tailings is essential to determine its quality for use as a material to conform geopolymers, the critical properties that must be monitored, and the potentially toxic elements existing in the waste.

First, a physical characterisation of the mining waste “Collado del Lobo” was carried out. The first of the tests was the UNE-EN 1097-7 particle density test. This test was carried out using the pycnometer method to determine the difference between the density of the waste and a conventional aggregate, 2.65 t/m^3^. These differences in density can cause homogenisation problems when mixing the waste with other materials, as well as in the transport and use of the geopolymer. For this reason, it is essential to determine the density. In addition, in order to evaluate the quality of the mine tailings for use in geopolymers, a test was carried out to determine the grading curve of the waste in accordance with the UNE-EN 933-1 standard. The particle size of the mining waste, determined by the mass passing through different sieves, has a significant influence on the quality of the geopolymer formed from it, as a lower particle size leads to a higher activation of the material and consequently to better mechanical and physical properties. To determine whether the particle size of the mine tailings was adequate, this test was carried out.

For the chemical characterisation of the material, an elemental analysis test was carried out. This test determines the percentage of carbon, nitrogen, and hydrogen in the waste, by analysing the gases produced during the ignition of the sample at 950 ± 5 °C. This test was performed with LECO’s TruSpec Micro equipment (TruSpec Micro, LECO, St. Joseph, MI, USA), in order to evaluate the presence of organic matter, nitrogen, carbon, etc., in the sample.

At the same time, and with the aim of evaluating the existence of volatile elements or the transformation of certain chemical compounds, a loss on ignition test was performed on the mine tailings sample at a temperature of 950 ± 5 °C. This test determines the variation in mass produced in the sample before and after subjecting it to the detailed temperature.

Finally, and with the aim to determine and quantify the existence of the elements of higher atomic weight, an X-ray fluorescence test was carried out with the ADVANT’XP+ commercial equipment (ADVANT’XP+, Thermo Fisher, Waltham, MA, USA). This test makes it possible to determine whether the mine tailings have an adequate composition of silicon and aluminium for geopolymer conformation, as well as the existence of potentially toxic elements that must be controlled throughout the process so that the conformed geopolymer does not produce unacceptable environmental contamination.

#### 2.2.2. Conforming of Samples: Physical and Mechanical Testing of Geopolymers

Once the waste was chemically and physically characterised, the different geopolymer families were conformed. As noted, the geopolymer is alkaline activated aluminosilicate from the mining tailings. Potassium hydroxide is the alkaline activator. Therefore, the appropriate proportion of both elements must be calculated in order to obtain a geopolymer with adequate physical and mechanical characteristics which, in addition, is capable of retaining the leaching of potentially toxic elements from the mining waste. The geopolymer families were conformed with mine tailings, 30% water, and increasing percentages of potassium hydroxide, as detailed in Table 1.

All the geopolymer families were conformed with the same procedure, making a total of nine samples per family to obtain statistically reliable results. For their manufacture, initially, the percentage of potassium hydroxide was diluted in water until it was correctly dissolved and, subsequently, this solution was mixed with the mine tailings until it was homogenised. The material thus prepared was poured into a metal matrix with internal dimensions of 60 mm in length and 30 mm in width, and then a pressure of 20 MPa was applied with a rammer of similar dimensions. The prepared samples were stripped and subjected to a temperature of 25 ± 2 °C for 24 h. Subsequently, they were dried at a temperature of 105 ± 2 °C for 24 h. This drying process is carried out because, as different authors have determined, the geopolymerisation temperature is very important. Therefore, the geopolymer is first subjected to ambient temperature so that the appropriate geopolymerisation reactions take place and, subsequently, it is dried at a higher temperature to eliminate water and stop all the reactions that have not been produced. It should be noted that the GC0 family has no percentage of alkaline activator, so no geopolymer will be formed. However, this family was developed to compare the results and to confirm that a geopolymerisation process was indeed taking place in the other families.

The conformed geopolymers from the different families were subjected to various physical tests. The first physical tests were the determination of the linear shrinkage and the weight loss that occurs in the material during the geopolymerisation process. The linear shrinkage and weight loss of the geopolymers was measured by quantifying the dimensions and weight of the samples after compaction and subsequently after the drying process. These tests, carried out in accordance with the UNE-EN 772-16 standard, are essential for the industrial use of this material, as it is commercially necessary for the product to have specific dimensions and weights. At the same time, in order to evaluate the effect of water on the geopolymer, the capillary water absorption (UNE-EN 772-11) and cold-water absorption (UNE-EN 772-21) tests were carried out. The capillary water absorption test is performed by partially immersing the sample in water and determining the difference in mass, and the cold-water absorption test is executed by immersing the sample in water for 24 h and determining the mass variation that occurs. Both tests evaluate the structure of the geopolymers and their capacity to absorb water, determining whether the geopolymers conformed have a greater or lesser open structure. In turn, the open porosity and apparent density of the different families of geopolymers were calculated according to the UNE-EN 772-4 standard. These tests, carried out by hydrostatic methods after subjecting the samples to boiling water, determine essential properties and determining factors for mechanical resistance and even other characteristics that are so important for a construction material, such as thermal and acoustic insulation.

The colour of the different geopolymer families was registered using a colourimeter, more specifically the commercial equipment PCE-RGB-2 (RGB-2, PCE, Meschede, Germany). This physical property, which is not regulated by standards, is very important for the marketing of a product. Therefore, within the subjectivity of the market itself, it can only be quantified and recorded on a scientific level.

Finally, the mechanical properties of the different geopolymers conformed were determined. This simple compressive strength test was carried out in accordance with the UNE-EN 772-1 standard, determining the strength of all the geopolymers and selecting on the basis of these results the one with the best mechanical properties, provided that the rest of the limitations established by the standard were met and acceptable results were obtained.

#### 2.2.3. Analysis of the Geopolymer Leachates

Finally, to evaluate the retention of potentially toxic elements in the mine tailings in the geopolymer matrix, the TCLP test was performed on all the geopolymer families. The TCLP test was selected as a method for leachate analysis because it is an international standard, applicable to different countries, and it is also used by several researchers to evaluate leachate in construction materials.

To perform the method, the geopolymers were ground to a particle size of less than 10 mm. Subsequently, this sample was mixed with the leaching solution at a solid/liquid ratio of 1:20 and, in turn, was agitated for 18 ± 2 h at a temperature of 22 ± 3 °C. The leach solution was composed of 5.7 mL of glacial acetic acid and 64.3 mL of sodium hydroxide solution (1 N) diluted in 1 L of distilled water. Subsequently, the solution after stirring was filtered with a glass fibre filter, effective pore size 0.7 μm, and acidified with nitric acid to a pH of 2. The extracted liquid was analysed by inductively coupled plasma mass spectrometry (7900, Agilent, Santa Clara, CA, USA).

In this way, the leaching of potentially toxic elements from the different geopolymer families could be estimated, establishing an adequate percentage of potassium hydroxide necessary for the correct encapsulation of the elements in the geopolymer.

## 3. Results

### 3.1. Physical and Chemical Characterisation of the Mine Tailings “Collado del Lobo”

First, to physically characterise the mine tailings, the density of the particles was calculated, obtaining a waste density of 2.62 ± 0.06 g/cm^3^. This density is very similar to that developed for conventional construction materials, which is around 2.65 g/cm^3^. Therefore, there should be no problems of homogenisation with other materials, nor should there be any special problems in their transport or manufacture of materials that would unnecessarily overload the structures made with them.

**Figure 1:**
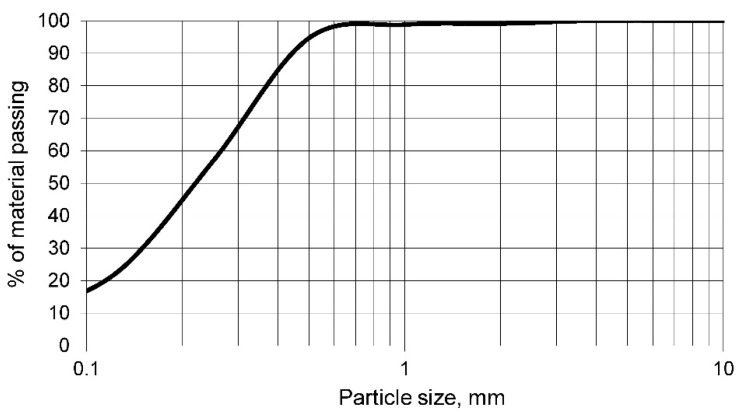
Grading curve of the mine tailings “Collado del Lobo”.

In turn, and as noted above, the particle size of the aluminosilicate has a significant influence on the quality of the geopolymer obtained. For this reason, the grading curve of the mine tailings was determined. This grading curve is shown in Figure 1.

The grading curve of the mine tailings shows that the highest percentage of particles has a size between 100 and 500 μm. Therefore, it shows that the particle size of the mine tailings can be considered as small and is suitable for geopolymer conforming. In addition, there are almost 20% of particles with a particle size smaller than 100 μm, so the geopolymerisation reaction will occur properly by creating a matrix that envelops the larger particles.

For the chemical characterisation, an elemental analysis test was initially carried out to determine the percentage of carbon, hydrogen, and nitrogen in the mine tailings sample. The results of this elemental analysis test are shown in Table 2.

The results of the elemental analysis test show that the percentage of nitrogen is very low. Consequently, there should be no environmental problems, or problems in the geopolymerisation process, with chemical compounds incorporating this element. In addition, the low percentage of hydrogen in relation to the percentage of carbon reflects the almost nonexistence of organic matter that could be detrimental to the geopolymer made. The percentage of carbon in the sample seems to correspond mainly to carbonate compounds, being equally low and not substantially altering the basic composition of an aluminosilicate.

Additionally, the loss on ignition test at a temperature of 950 ± 5 °C shows that the variation in mass before and after subjecting the sample to this temperature is 6.89 ± 0.25%. This percentage is very low, which demonstrates the quality of the mine tailings as aluminosilicate, as it reflects the low proportion of volatile elements and carbonates that could damage the geopolymerisation process and assume a useless load in the material.

Finally, in order to determine the elements with the highest atomic weight in the tailings, an X-ray fluorescence test was carried out, obtaining the results shown in Table 3.

The X-ray fluorescence test shows that the mine tailings are suitable for use as a source of aluminosilicates for the manufacture of geopolymers. This is because the percentage of silicon is high and that of aluminium is considerable, with a ratio between them of approximately 5. Calcium, potassium, iron, and sodium appear in smaller but considerable proportions, being common elements in the mine tailings of the district evaluated. It is worth noting the existence of chemical elements that are found in smaller proportions but can cause environmental contamination; these elements are called potentially toxic. Among these elements, the one with the highest percentage in the sample is lead, a chemical element extracted by the old mining operations. Barium, in turn, also exists in the sample and, like the previous chemical element, must be controlled to prevent the geopolymer conformed with the mine tailings from producing contaminating leachates of both elements. The other elements controlled by the EPA for this type of construction material—arsenic, cadmium, and chromium—were found in very low proportions. However, they will also be assessed by leaching to confirm that they do not cause any environmental problems.

### 3.2. Conforming of Samples: Physical and Mechanical Testing of Geopolymers

Once the mine tailings were physically and chemically analysed, the different families of geopolymers detailed in Table 1 were conformed according to the procedure described in the methodology. These families of samples were subjected to different physical and mechanical tests.

**Figure 2:**
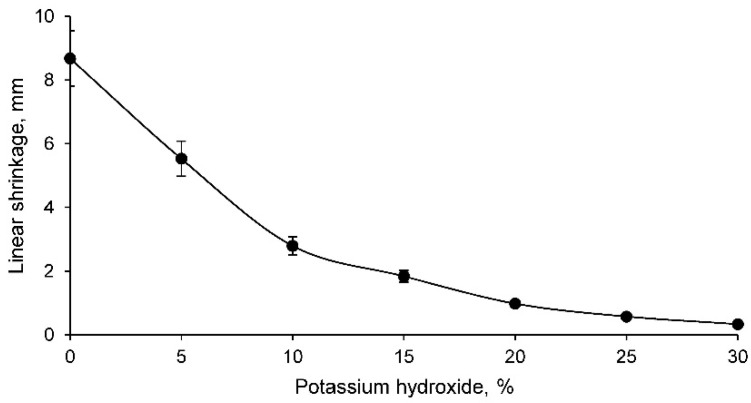
Linear shrinkage of different geopolymer families.

The first of the physical tests was the determination of linear shrinkage, with the results shown in Figure 2 as a function of the percentage of potassium hydroxide.

The linear shrinkage of the conformed geopolymers decreases as the percentage of potassium hydroxide increases. This fact shows that the samples conformed with low percentage of potassium hydroxide do not adequately retain their shape and significant dimensional variations occur during the geopolymerisation process. This is to be expected if one considers that the 0% potassium hydroxide family is composed only of mine tailings and is therefore only a compacted material. The samples with percentages higher than 15% undergo less linear shrinkage, maintaining more of their shape and with less variation between results.

**Figure 3:**
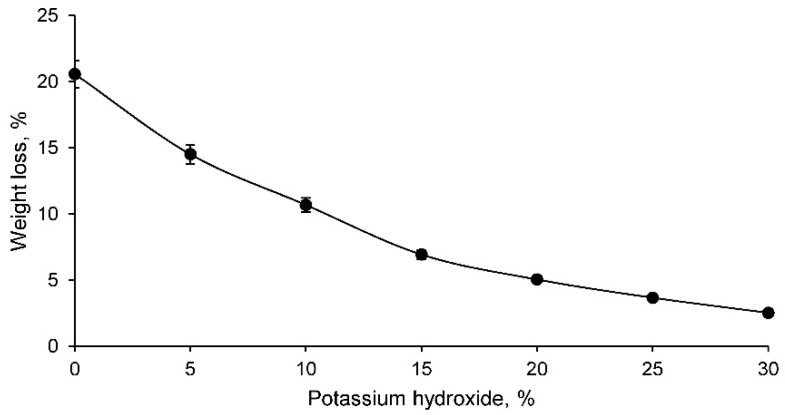
Weight loss of the different geopolymer families.

The weight loss test results of the different geopolymer families are shown in Figure 3.

The weight loss is lower in the families containing higher percentages of potassium hydroxide. This fact reflects, as with linear shrinkage, that families with low potassium hydroxide percentage have lower capacity to conserve their shape and mass during the geopolymerisation process. Consequently, it can be stated that they are lower quality families due to the fact that a correct geopolymerisation process has not taken place. Families with more than 15% of potassium hydroxide have a smaller variation of results, showing acceptable values.

**Figure 4:**
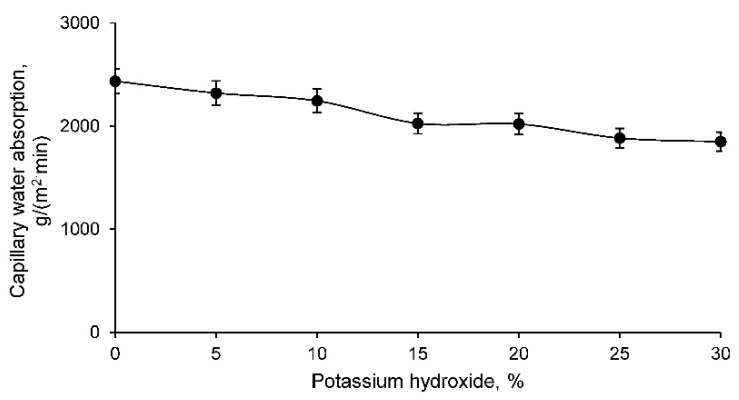
Capillary water absorption of different geopolymer families.

The capillary water absorption test reflects the capacity of a material to absorb water when it is semi-submerged. This test produced the results shown in Figure 4 for all geopolymer families conformed.

The capillary water absorption of the geopolymer families conformed with the mine tailings is lower with higher percentage of potassium hydroxide. As can be interpreted from the results, families with a higher percentage of potassium hydroxide have a more closed structure and a smaller number of interconnected pores, resulting in less water suction through this structure. This property is essential to be evaluated in building materials that are exposed to the water table or to the soil’s own humidity, in which case a lower absorption of water by capillarity is of interest.

**Figure 5:**
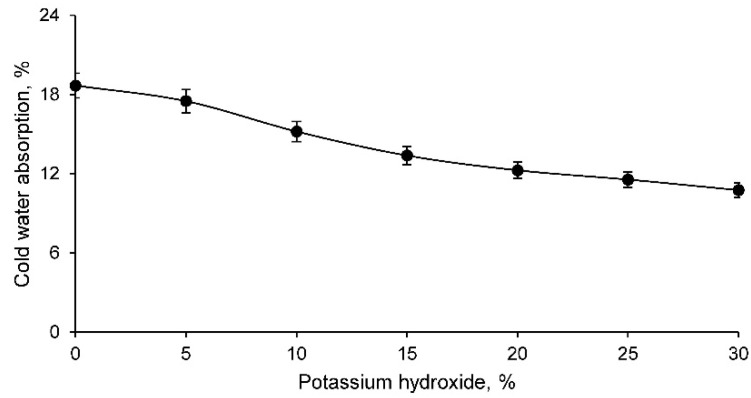
Cold water absorption of different geopolymer families.

The cold-water absorption test results are shown in Figure 5 for all geopolymer families.

The absorption of cold water, as with capillary water absorption, is lower with higher percentage of potassium hydroxide in the geopolymer. This shows that the structure of the material is more closed and possibly has fewer pores, making it more difficult to absorb water and, consequently, to vary the mass. Therefore, this test is essential for those building materials that are outdoors, as they are exposed to rainwater and, consequently, can absorb this water. This water absorption therefore increases the weight of the material and unnecessarily overloads the structure that supports the materials. Nevertheless, the results obtained are acceptable, as they are similar to those developed for conventional construction materials.

**Figure 6:**
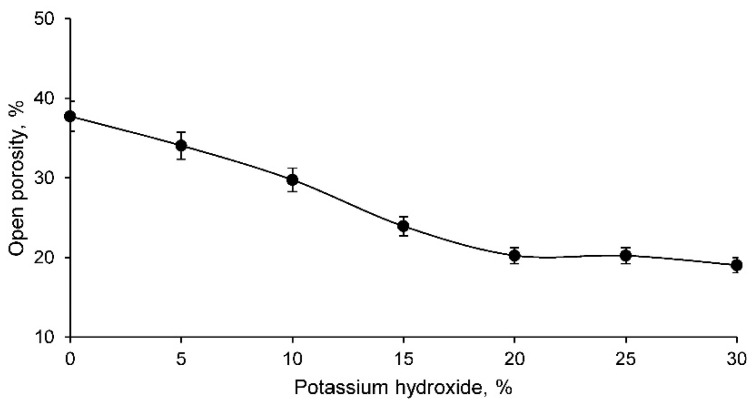
Open porosity of the different geopolymer families.

The open porosity of the different families of geopolymers conformed is shown in Figure 6.

The open porosity, as expected from the results obtained for capillary water absorption and cold-water absorption, is lower with higher percentage of potassium hydroxide in the geopolymer. This fact reflects that there is a lower percentage of pores in the geopolymers with a higher percentage of potassium hydroxide, developing a more closed structure and, consequently, more resistant under equal conditions. However, the strength will be evaluated through the corresponding test.

**Figure 7:**
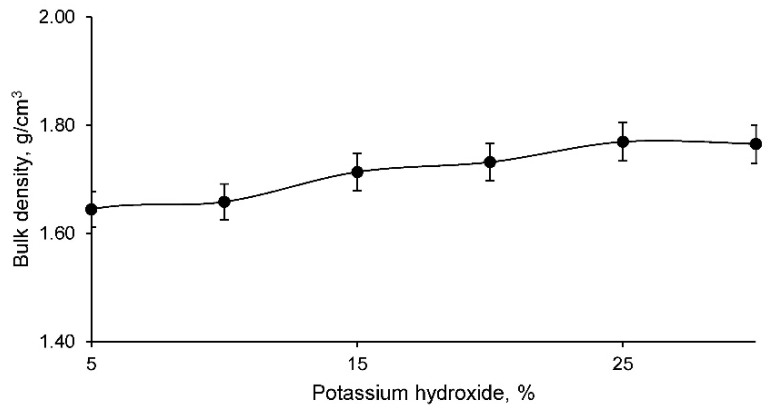
Bulk density of the different geopolymer families.

Bulk density, in turn, was evaluated for all geopolymer families, with the results shown in Figure 7.

The bulk density increases as the percentage of potassium hydroxide increases. This is to be expected based on the previous porosity results. Therefore, as noted above, a more compact material was developed with increasing potassium hydroxide percentage. However, the densities obtained appear to be slightly lower than those of other construction materials such as ceramics, mortar, concrete, etc. This lower density will influence the strength, but also possibly develop interesting properties such as thermal or acoustic insulation.

**Figure 8:**
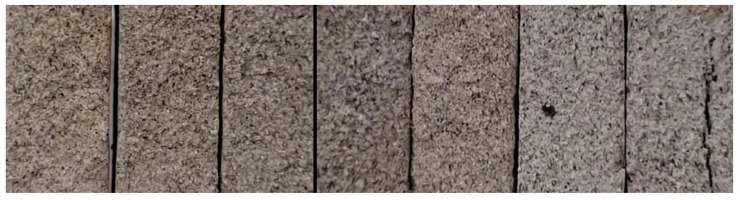
Image of the geopolymers conformed from the different sample families. From left to right: GC0, GC5, GC10, GC15, GC20, GC25, and GC30.

As detailed above, colour is not a physical property limited by regulations. However, it is a characteristic that is highly controlled by the companies producing construction materials, as the materials must have a suitable colour to be marketed. It is therefore necessary to quantify and registered the colour of geopolymers scientifically. Figure 8 shows the different families of samples conformed with different percentages of potassium hydroxide and, in Table 4, the colour coordinates of each of these families are registered.

**Figure 9:**
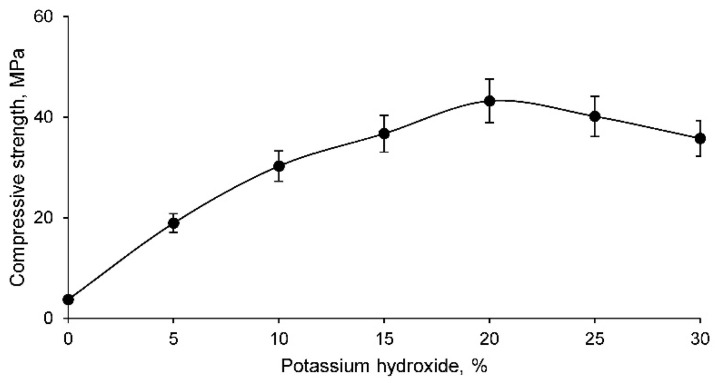
Simple compressive strength of the different geopolymer families.

Finally, because the geopolymer is intended for a construction material, the simple compressive strength must be calculated. The simple compressive strength test results for all geopolymer families are shown in Figure 9.

The simple compressive strength of the geopolymers increases as the percentage of potassium hydroxide increases, exhibiting a maximum strength around 20% potassium hydroxide and decreasing thereafter. The practically zero resistance of the family with 0% potassium hydroxide was to be expected if one considers that no geopolymer was formed, since there is no alkaline activator and it only consists of a compacted material. This fact confirms that in the successive percentages, geopolymers have been formed, as they have higher strengths due to the addition of the alkaline activator. In contrast, the families with lower percentages of potassium hydroxide had lower density and higher porosity, so their lower strength is justifiable. However, the families with higher percentages of potassium hydroxide showed higher density and lower porosity, so the decrease in strength is not equally justifiable. However, it should be noted that the families with 25% potassium hydroxide after the geopolymerisation process showed small cracks on the surface, this percentage of cracks being much higher in the family with 30% potassium hydroxide. Therefore, this cracking of the material, due to a high percentage of potassium hydroxide that cannot absorb and react with the mine tailings, results in a lower simple compressive strength. Consequently, the most suitable geopolymer families for use, with acceptable and considerable strengths, are those with a percentage of potassium hydroxide of around 20%.

### 3.3. Analysis of the Geopolymer Leachates

The aim of this research is to prevent mining tailings, which mainly contain heavy metals, from causing environmental pollution. Therefore, this waste was used as aluminosilicate for the conformation of geopolymers, activating this element with different percentages of potassium hydroxide. These geopolymers have been physically and mechanically evaluated, determining their suitability for use. However, it is essential to corroborate that the contaminating elements in the mine tailings do not produce a leachate with high concentrations of these elements. For this purpose, the TCLP test was developed for all geopolymer families. The leachates thus produced must comply with the limitations established by the EPA for the chemical elements under study. These limits are shown in Table 5.

**Figure 10:**
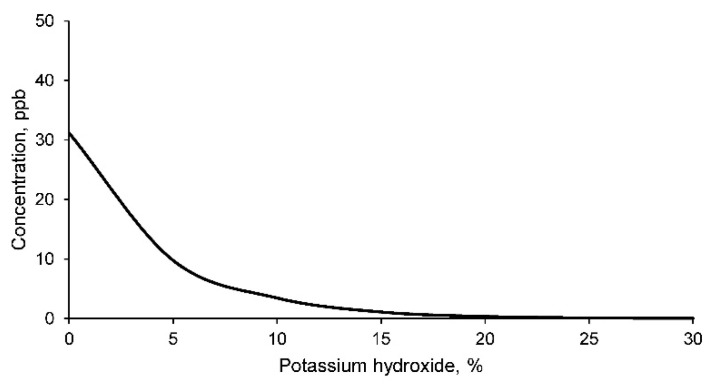
Chrome concentration in the leachates of geopolymers conformed with different percentages of potassium hydroxide according to the TCLP test method.

The TCLP test results, performed on all conformed geopolymer families, reflect the chromium leachate concentrations shown in Figure 10.

The concentration of chrome in the leachate decreases as the percentage of potassium hydroxide increases, which is to be expected if one considers that the geopolymer is being formed and, therefore, its matrix retains the contaminating elements. It should be noted that all the concentrations of chrome in the leachate are lower than the regulatory limit for this element, 5000 ppb, even for the tailings sample without any type of treatment. Therefore, the mine tailings sample does not produce contaminating leachate with respect to chrome, although it is true that the concentration of chrome in the leachate decreases as the percentage of potassium hydroxide increases.

**Figure 11:**
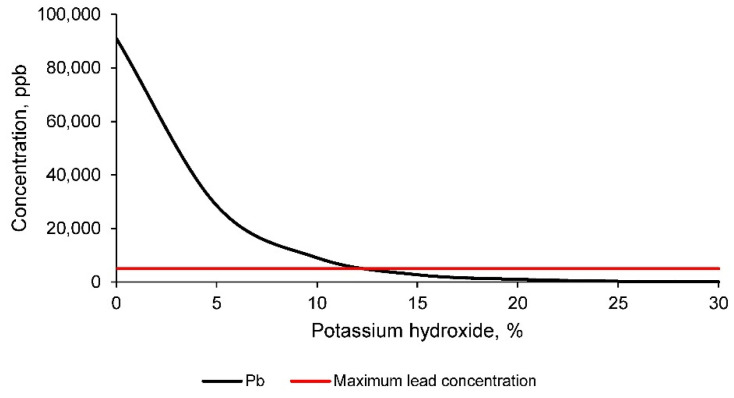
Lead concentration in the leachates of geopolymers conformed with different percentages of potassium hydroxide according to the TCLP test method.

The concentrations of lead in the leachate according to the TCLP method for the different geopolymer families are shown in Figure 11.

As can be seen, the concentration of lead in the leachate of the mine tailings sample is higher than the concentration limited by the EPA. Consequently, it can be affirmed that this mine tailing is producing environmental contamination with respect to this element. In turn, it should be noted that the concentration of lead in the leachate decreases as the percentage of potassium hydroxide increases, obtaining acceptable values according to the EPA standard in geopolymers with a percentage higher than 15% of potassium hydroxide. Consequently, geopolymer families with percentages above 15% potassium hydroxide are acceptable for use as construction materials.

**Figure 12:**
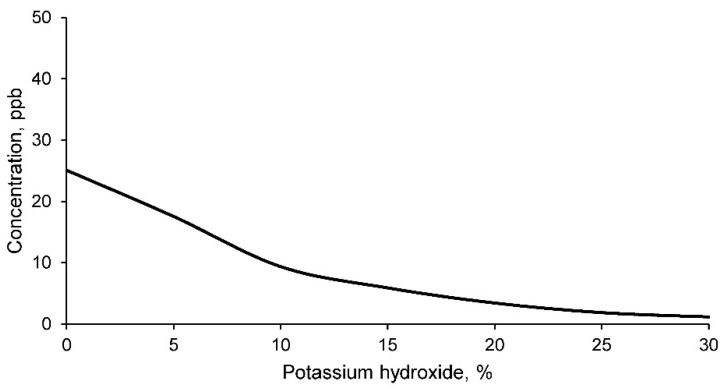
Arsenic concentration in the leachates of geopolymers conformed with different percentages of potassium hydroxide according to the TCLP test method.

In turn, the arsenic concentration in the leachate of all geopolymer families is shown in Figure 12.

The concentration of arsenic in the leachate of the geopolymers is in all cases below the regulatory limits for use as building materials. However, it should be noted that this concentration decreases substantially to negligible minima as the geopolymer is formed with increasing percentage of potassium hydroxide.

**Figure 13:**
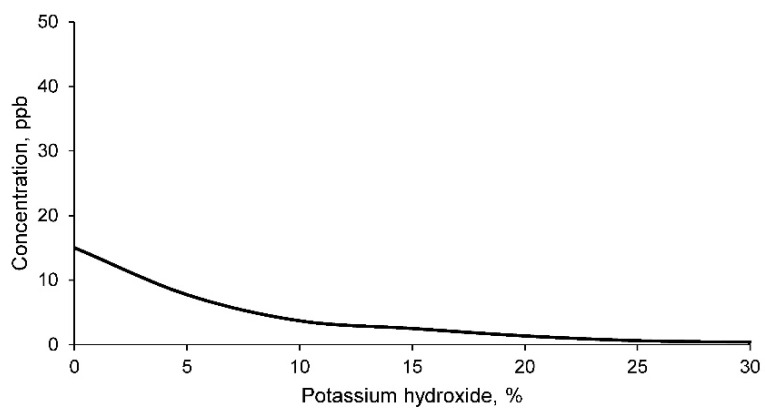
Cadmium concentration in the leachates of geopolymers conformed with different percentages of potassium hydroxide according to the TCLP test method.

The cadmium concentration in the leachate according to the TCLP test method for all geopolymer families is shown in Figure 13.

The concentration of cadmium in the geopolymer leachate, as is the case for chrome and arsenic, is in all geopolymer families lower than the limits set by the EPA. Moreover, mine tailings themselves do not produce environmental contamination, according to the limitations noted above, for this chemical element. However, as in all previous cases, the concentration of cadmium decreases as the percentage of potassium hydroxide increases, demonstrating that the formation of the geopolymer retains the leaching of this pollutant element.

**Figure 14:**
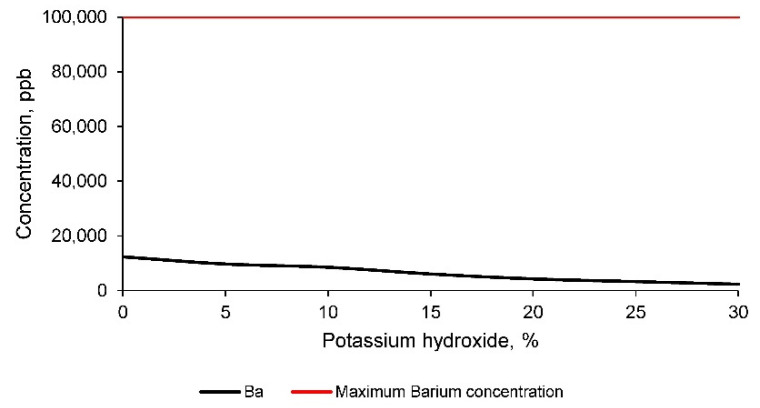
Barium concentration in the leachates of geopolymers conformed with different percentages of potassium hydroxide according to the TCLP test method.

Finally, the concentration of barium in the leachate of geopolymers conformed with mine tailings and different percentages of potassium hydroxide is evaluated. These results are shown in Figure 14.

The concentration of barium in all the leachates is lower than the limits set by the EPA. Consequently, it can be affirmed that the mine tailings do not produce environmental contamination according to the restrictions detailed by the regulations and with respect to this chemical element. However, it should be noted that the concentration of barium in the leachate decreases as the percentage of potassium hydroxide increases. Therefore, it can be considered that the formation of the geopolymer retains the leaching of this element, as was the case for all the chemical elements evaluated.

## 4. Conclusions

The results of the tests noted in the methodology allow a series of partial conclusions to be drawn that lead to the confirmation of the final objective. Therefore, partial conclusions are detailed below.

- Mine tailings have a similar density to any conventional aggregate. At the same time, the particle size of this tailings was mostly smaller than 500 μm. Consequently, the geopolymerisation process can be carried out adequately, as was corroborated by different authors.
- The chemical composition of the tailings showed a low proportion of organic matter, as there were no high percentages of carbon and hydrogen. In addition, the low percentage of loss on ignition reflected the low proportion of volatile elements and carbonates.
- The X-ray fluorescence test showed that the mine tailings were suitable for use as aluminosilicate, as the elements with the highest proportion in the sample were silicon and aluminium, both elements having a ratio of approximately 5. In addition, potentially toxic elements such as barium, cadmium, chrome, arsenic, etc., were found in the tailings. However, the potentially toxic element with the highest proportion, and the most limiting for the use of this waste as a geopolymer, was lead.
- The linear shrinkage, weight loss, capillary water absorption, cold-water absorption, and open porosity of the geopolymers conformed with the tailings decrease as the percentage of potassium hydroxide increases. Thus, it was shown that the geopolymer is conforming with higher percentages of potassium hydroxide and that it had a more closed structure with a lower percentage of pores. This fact was also reflected in the bulk density, which decreased with increasing percentage of potassium hydroxide.
- The mechanical strength of the different geopolymers conformed increased with increasing percentage of potassium hydroxide up to a maximum of 20%, after which this strength decreased.
- The concentration of potentially toxic elements, evaluated by the EPA in the leachate obtained according to the TCLP test, decreased as the percentage of potassium hydroxide increased, showing that the geopolymer is being conformed and that this material retained the contaminating elements. The chemical elements chrome, cadmium, arsenic, and barium do not present environmental contamination from the mine tailings. However, it was found that mine tailings can leach unacceptable concentrations of lead and that these concentrations decreased in the leachate of geopolymers conformed with a percentage of potassium hydroxide higher than 15%.

Consequently, the results obtained show that it is possible to formulate geopolymers with the mining tailings “Collado del Lobo” and potassium hydroxide. The best mechanical properties are obtained with the mining tailings, 30% mixing water, and 20% potassium hydroxide, obtaining adequate physical properties of this family for the use of the developed material as a construction material. In addition, the family conformed with the detailed percentages, avoids the leaching of lead in unacceptable concentrations according to the EPA, therefore avoiding the environmental contamination produced by this element. Consequently, this research develops a new material for construction with mainly mining waste, avoids the environmental pollution that can be produced by this mining waste, reduces the extraction of raw materials, and the deposition in waste dumps, and also creates a sustainable material applicable for construction.

## Figures and Tables

**Table 1:** Family of geopolymers conformed with the mine tailings “Collado del Lobo” and different percentages of potassium hydroxide.

Family	Potassium Hydroxide, %
GC0	0
GC5	5
GC10	10
GC15	15
GC20	20
GC25	25
GC30	30

**Table 2:** Elemental analysis of mine tailings.

Sample	Nitrogen, %	Carbon, %	Hydrogen, %
Mine tailings	0.151 ± 0.003	2.244 ± 0.057	0.074 ± 0.002

**Table 3:** X-ray fluorescence of mine tailings.

Element	wt.%	Est. Error
Si	29.24	0.11
Al	5.75	0.08
Ca	5.28	0.09
K	3.64	0.09
Fe	1.79	0.06
Na	1.19	0.05
Pb	0.981	0.05
Mg	0.525	0.026
Ba	0.687	0.05
Ti	0.161	0.0080
Sx	0.103	0.0051
Mn	0.173	0.0086
Other elements	0.2051	0.0027

**Table 4:** Colour coordinates (red, green, and blue) of the different conformed geopolymer families.

Sample	R	G	B
GC0	489	419	351
GC5	524	450	382
GC10	470	416	360
GC15	434	385	337
GC20	423	373	323
GC25	489	446	390
GC30	500	456	408

**Table 5:** Maximum concentrations of metals or toxic elements in the leachate according to the TCLP method (U.S. EPA).

Metals	Maximum Allowable Concentration in the Leachate, ppb
Cr	5000
Pb	5000
As	5000
Cd	1000
Ba	100,000

## Data Availability

Data are contained within the article.

## References

[1] Wu X.F., Chen G.Q. (2017). Global primary energy use associated with production, consumption and international trade. Energy Policy.

[2] Hartley K., van Santen R., Kirchherr J. (2020). Policies for transitioning towards a circular economy: Expectations from the European Union (EU). Resour. Conserv. Recycl..

[3] Kinnunen P.H.M., Kaksonen A.H. (2019). Towards circular economy in mining: Opportunities and bottlenecks for tailings valorization. J. Clean. Prod..

[4] Almeida M.I., Dias A.C., Demertzi M., Arroja L. (2015). Contribution to the development of product category rules for ceramic bricks. J. Clean. Prod..

[5] Carmo F.F., Lanchotti A.O., Kamino L.H.Y. (2020). Mining Waste Challenges: Environmental Risks of Gigatons of Mud, Dust and Sediment in Megadiverse Regions in Brazil. Sustainability.

[6] Mancini L., Sala S. (2018). Social impact assessment in the mining sector: Review and comparison of indicators frameworks. Resour. Policy.

[7] Souza-Filho P.W.M., Cavalcante R.B.L., Nascimento W.R., Nunes S., Gastauer M., Santos D.C., Silva R.O., Sahoo P.K., Salomão G., Silva M.S. (2020). The sustainability index of the physical mining Environment in protected areas, Eastern Amazon. Environ. Sustain. Indic..

[8] González-Martínez A., de Simón-Martín M., López R., Táboas-Fernández R., Bernardo-Sánchez A. (2019). Remediation of Potential Toxic Elements from Wastes and Soils: Analysis and Energy Prospects. Sustainability.

[9] Zhang L., Liu B., Du J., Liu C., Wang S. (2019). CO_2_ emission linkage analysis in global construction sectors: Alarming trends from 1995 to 2009 and possible repercussions. J. Clean. Prod..

[10] Oti J.E., Kinuthia J.M. (2012). Stabilised unfired clay bricks for environmental and sustainable use. Appl. Clay Sci..

[11] Raja V.K.B., Raj S.K., Sairam M.D., Kasyap A.V.R.S., Kumar V.G., Padmapriya R., Baalamurugan J., Sonawane P.D. (2021). Geopolymer green technology. Mater. Today Proc..

[12] Suvorova O.V., Selivanova E.A., Mikhailova J.A., Masloboev V.A., Makarov D.V. (2020). Ceramic Products from Mining and Metallurgical Waste. Appl. Sci..

[13] Terrones-Saeta J.M., Suárez-Macías J., Castañón A.M., Corpas-Iglesias F.A. (2021). Evaluation of Copper Leaching for Subsequent Recovery from the Waste Dumps of the Linares Mining District and Their Use for Construction Materials. Metals.

[14] Suárez-Macías J., Terrones-Saeta J.M., Iglesias-Godino F.J., Corpas-Iglesias F.A. (2020). Retention of Contaminants Elements from Tailings from Lead Mine Washing Plants in Ceramics for Bricks. Minerals.

[15] Terrones-Saeta J.M., Suárez-Macías J., Iglesias-Godino F.J., Corpas-Iglesias F.A. (2020). Study of the Incorporation of Biomass Bottom Ashes in Ceramic Materials for the Manufacture of Bricks and Evaluation of Their Leachates. Materials.

[16] Turner L.K., Collins F.G. (2013). Carbon dioxide equivalent (CO2-e) emissions: A comparison between geopolymer and OPC cement concrete. Constr. Build. Mater..

[17] Davidovits J. (1989). Geopolymers and geopolymeric materials. J. Therm. Anal..

[18] Singh N.B., Middendorf B. (2020). Geopolymers as an alternative to Portland cement: An overview. Constr. Build. Mater..

[19] Terrones-Saeta J.M., Suárez-Macías J., Iglesias-Godino F.J., Corpas-Iglesias F.A. (2021). Development of Geopolymers as Substitutes for Traditional Ceramics for Bricks with Chamotte and Biomass Bottom Ash. Materials.

[20] Duxson P., Fernández-Jiménez A., Provis J.L., Lukey G.C., Palomo A., van Deventer J.S.J. (2006). Geopolymer technology: The current state of the art. J. Mater. Sci..

[21] Sellami M., Barre M., Toumi M. (2019). Synthesis, thermal properties and electrical conductivity of phosphoric acid-based geopolymer with metakaolin. Appl. Clay Sci..

[22] Mudgal M., Singh A., Chouhan R.K., Acharya A., Srivastava A.K. (2021). Fly ash red mud geopolymer with improved mechanical strength. Clean. Eng. Technol..

[23] Lancellotti I., Kamseu E., Michelazzi M., Barbieri L., Corradi A., Leonelli C. (2010). Chemical stability of geopolymers containing municipal solid waste incinerator fly ash. Waste Manag..

[24] Bankowski P., Zou L., Hodges R. (2004). Reduction of metal leaching in brown coal fly ash using geopolymers. J. Hazard. Mater..

[25] Carrillo-Beltran R., Corpas-Iglesias F.A., Terrones-Saeta J.M., Bertoya-Sol M. (2021). New geopolymers from industrial by-products: Olive biomass fly ash and chamotte as raw materials. Constr. Build. Mater..

[26] Kumar A., Kumar S. (2013). Development of paving blocks from synergistic use of red mud and fly ash using geopolymerization. Constr. Build. Mater..

[27] Ferone C., Colangelo F., Roviello G., Asprone D., Menna C., Balsamo A., Prota A., Cioffi R., Manfredi G. (2013). Application-Oriented Chemical Optimization of a Metakaolin Based Geopolymer. Materials.

[28] Hattaf R., Aboulayt A., Samdi A., Lahlou N., Touhami M.O., Gomina M., Moussa R. (2021). Reusing Geopolymer Waste from Matrices Based on Metakaolin or Fly Ash for the Manufacture of New Binder Geopolymeric Matrices. Sustainability.

[29] Hertel T., Pontikes Y. (2020). Geopolymers, inorganic polymers, alkali-activated materials and hybrid binders from bauxite residue (red mud)—Putting things in perspective. J. Clean. Prod..

[30] Nath S.K. (2020). Fly ash and zinc slag blended geopolymer: Immobilization of hazardous materials and development of paving blocks. J. Hazard. Mater..

[31] Luhar S., Cheng T.W., Nicolaides D., Luhar I., Panias D., Sakkas K. (2019). Valorisation of glass waste for development of Geopolymer composites—Mechanical properties and rheological characteristics: A review. Constr. Build. Mater..

[32] Toniolo N., Rincón A., Avadhut Y.S., Hartmann M., Bernardo E., Boccaccini A.R. (2018). Novel geopolymers incorporating red mud and waste glass cullet. Mater. Lett..

[33] Yadav A.L., Sairam V., Srinivasan K., Muruganandam L. (2020). Synthesis and characterization of geopolymer from metakaolin and sugarcane bagasse ash. Constr. Build. Mater..

[34] Nkwaju R.Y., Djobo J.N.Y., Nouping J.N.F., Huisken P.W.M., Deutou J.G.N., Courard L. (2019). Iron-rich laterite-bagasse fibers based geopolymer composite: Mechanical, durability and insulating properties. Appl. Clay Sci..

[35] Saba M., Fakhari-Tehrani F., Michaud P., Hajikarimi P., Absi J. (2021). Experimental and Numerical Investigation of Sodium- and Potassium-Based Alkali Activator on the Mechanical Properties of Geopolymer-Mortars Using Lebanese Kaolin. Int. J. Civ. Eng..

[36] Provis J.L., Van Deventer J.S.J. (2009). Introduction to geopolymers. Geopolym. Struct. Process. Prop. Ind. Appl..

